# Toward a More Comprehensive Approach for Dissolved Organic Matter Chemical Characterization Using an Orbitrap Fusion Tribrid Mass Spectrometer Coupled with Ion and Liquid Chromatography Techniques

**DOI:** 10.1021/acs.analchem.3c02599

**Published:** 2024-02-19

**Authors:** Daniela Bergmann, Jessie Matarrita-Rodríguez, Hussain Abdulla

**Affiliations:** † Department of Physical and Environmental Sciences, Texas A&M University-Corpus Christi , Corpus Christi, Texas 78412, United States; ‡ Center for Water Supply Studies, Texas A&M University-Corpus Christi , Corpus Christi, Texas 78412, United States; § Centro de Investigación en Contaminación Ambiental (CICA), Universidad de Costa Rica, San José 11501-2060, Costa Rica

## Abstract

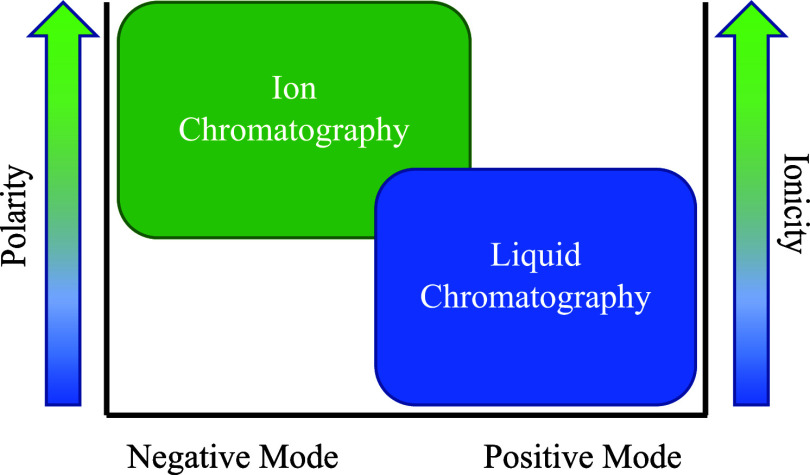

Dissolved organic matter (DOM) represents one of the largest active organic carbon pools in the global carbon cycle. Although extensively studied, only <10% of DOM has been chemically characterized into individual dissolved compounds due to its molecular complexity. This study introduced a more comprehensive DOM characterization method by coupling both ion chromatography (IC) and liquid chromatography (LC) with high mass accuracy and resolution mass spectrometry. We presented a new *on-the-fly* mass calibration of the Orbitrap technique by utilizing the “lock mass” function in the Orbitrap Fusion Tribrid mass spectrometer (OT-FTMS), which assures high mass accuracy at every scan by a postcolumn introduction of internal labeled standards. With both IC and LC, tested unlabeled standards of amino acids, small peptides, and organic acids were consistently below 1.0 ppm mass error, giving the OT-FTMS the potential of reaching mass accuracy of the Fourier-transform ion cyclotron resonance mass spectrometer. In addition to mass accuracy, a pooled quality control sample (QC) was used to increase reproducibility by applying systematic error removal using random forest (SERRF). Using an untargeted mass spectrometry approach, estuarine DOM samples were analyzed by OT-FTMS coupled to IC in negative mode and LC in positive mode detection to cover a wide range of highly cationic to highly anionic molecules. As a proof of concept, we focused on elucidating the structures of three distinct DOM compound classes with varied acidities and basicities. In UPLC-OT-FTMS, a total of 915 compounds were detected. We putatively elucidated 44 small peptides and 33 deaminated peptides of these compounds. With IC-OT-FTMS, a total of 1432 compounds were detected. We putatively elucidated 20 peptides, 268 deaminated peptides, and 188 organic acids. Except for five compounds, all putatively elucidated compounds were uniquely detected in their corresponding chromatography technique. These results highlight the need for combining these two techniques to provide a more comprehensive method for DOM characterization. Application of the combined IC and LC techniques is not limited to DOM chemical characterization. It can analyze other complex compound mixtures, such as metabolites, and anthropogenic pollutants, such as pesticides and endocrine-disrupting chemicals, in environmental and biological samples.

## Introduction

Dissolved organic matter (DOM) makes up around 90% of the total marine organic carbon and is one of the largest active organic carbon pools in the global carbon cycle.^1−4^ It consists of a complex mixture of thousands of organic compounds with diverse molecular compositions and various chemical functional groups.^2,5,6^ To understand and quantify the significance of the different DOM biogeochemical processes and sources in aquatic ecosystems, we must decipher the chemical codes imprinted by these processes on the chemical structures of the DOM pool. However, the complex chemical nature of DOM makes it one of the most challenging natural samples to analyze at a molecular structure level, leaving most DOM compounds still unidentified.^7−9^

The recent advances in high-resolution accurate mass spectrometry (HR-AM) like Fourier-transform ion cyclotron resonance (FT-ICR-MS) open the opportunity to analyze DOM at the molecular level. However, its relatively slow acquisition rate has hindered FT-ICR-MS from taking full advantage of online coupling to different chromatography techniques. On the other hand, the recent introduction of a new type of HR-AM spectrometer, the Orbitrap mass spectrometer, allowing a faster acquisition rate, is a requirement for successful coupling with chromatography techniques.^9−11^ To achieve the structural elucidation and quantification of individual DOM compounds, the mass spectrometer analysis method is required to (1) reduce the DOM complexity and separate different structural DOM isomers, (2) measure the mass of wide varieties of individual compounds and with high resolution and mass accuracy, (3) generate tandem fragmentation spectra for the majority of individual DOM compounds for structural elucidation, and (4) provide high analysis reproducibility to identify the significant differences in DOM chemical composition at different ecosystems.

Most previous studies have used electrospray ionization (ESI) direct injection to FT-ICR-MS or Orbitrap mass spectrometer to analyze DOM in negative mode.^10^ Recently, ultraperformance liquid chromatography (UPLC) techniques have been utilized in combination with negative and positive ESI modes for the analysis of DOM, including reversed-phase C18 (RP) and hydrophilic-interacting liquid chromatography (HILIC).^12,13^ However, these chromatography techniques are established on silica-based columns that cannot handle higher pH mobile phases,^14,15^ which overlook detecting compounds that have low ionization efficiency at low pH, like organic acids. The UPLC techniques have been mainly optimized for positive mode detection (for amine or other functional groups that can be ionized by protonation). In HILIC, the column separation efficiency decreases dramatically with increasing injection volume and many studies use an injection volume no higher than 5 μL.^14^ Recently, ion chromatography (IC) application in ESI negative mode has shown promising better separation and improved sensitivity of highly polar and anionic compounds compared to HILIC.^15,16^ This makes IC (in negative mode) an ideal complement to UPLC in positive mode to detect various DOM compounds comprehensively.

Given the complexity of DOM, enhancing the mass accuracy to subppm levels reduces the potential molecular formula candidates, facilitating the confident assignment of detected mass features from various DOM compounds to specific molecular formulas.^17,18^ Accurate calibration of each mass spectrum along the entire chromatogram is crucial for the correct molecular formula assignment. While many approaches, including using UPLC background ions as a “lock mass” and spiked labeled compounds (e.g., caffeine) in the mobile phase, have been used for internal calibration,^19−25^ they often suffer from inconsistent signal intensities and retention time shifts. Another approach is using the optional features of dual sprayer ionization in specific mass spectrometers to introduce locking internal standards in the electrospray chamber. However, it can be expensive^26^ and result in analyte gaps when switching between sample and reference spray.^27,28^ Other postcolumn strategies include using a six-port valve^26^ or adding a T-joint connector that mixes the analyte flow with the reference flow and has proven efficient, providing a consistent flow rate and signal.^27,29^ In this study, we used a T-joint connector to introduce a modified mass locking technique called “*on-the-fly* calibration” that has flexibility in choosing various internal labeled standards according to the experiment’s needs.

To identify the significant differences between analyzed samples, QC samples are used to control the instrument’s intra- and interday precision, reduce instrumental errors, and increase sample analysis reproducibility.^30^ One of the traditional strategies is to spike internal standards into samples to minimize variations in the analysis. Still, this technique can be time-consuming and expensive if done for hundreds of samples.^31^ Additionally, it is based on the assumption that the internal standard represents the whole sample batch, which cannot be applied to complex mixtures such as metabolites or DOM.^32^ Another strategy is using different sample normalization approaches to reduce technical errors between samples, such as total sum normalization,^33^ median normalizations,^34^ or constant sum.^35^ An alternative strategy is using a pooled QC sample approach, a mixture of all samples, which has become a new standard method in untargeted analysis to observe analytical accuracy and repeatability and, if necessary, correct any variations between and within runs.^31,36^ Recently, Fan et al. introduced a random forest-based package called systematic error removal using random forest (SERRF) based on utilizing pooled QC samples to remove drifts and intercorrelated error within sample runs and they outperformed compared to traditional normalization approaches of mass spectrometer data and reduced the average technical errors to 5% relative standard deviation.^37^ This study will evaluate and compare constant sum normalization to the SERRF on a set of DOM samples.

This study aims to introduce a more comprehensive approach to DOM chemical characterization using an Orbitrap Fusion Tribrid Mass spectrometer (OT-FTMS) coupled with an IC and UPLC for untargeted mass spectrometry analysis. This approach will enhance the identification of DOM compounds with various physicochemical characteristics in environmental samples and cover both positive and negative ionization modes. Second, by continuously supplying labeled internal standards *(on-the-fly),* the OT-FTMS can apply a “lock mass” during the run to reduce overall mass error, comparable to the FT-ICR-MS mass accuracy. Third, any intra- or interday variations of the mass spectrometer will be corrected by applying random forest statistical analysis on QC samples that were run between samples.

## Experimental Section

### Sample Preparation and Chemical Standards

A total of 36 surface water samples were collected in 1 L precleaned polycarbonate bottles at 18 stations (in duplicates) in Nueces Bay, Texas, from September 07–25, 2022 (Figure S1). Surface water samples were sterile filtered using Corning disposable sterile Bottle-Top filters with a 0.22 μm membrane. The sterile filtered samples were acidified to pH 2 with trace metal grade hydrochloric acid (Thermo Scientific) and processed using solid-phase extraction (1g, 6 mL Bond Elut-PPL cartridges) according to Dittmar et al. with modification^7^ (see the SI for details). A quality control (QC) pool sample was made by combining an aliquot of 50 μL from all the samples into one vial, including porewater and nepheloid layer samples that were not discussed in this paper.

Mixtures of 17 unlabeled organic acid standards (Table S1) were used for evaluating *on-the-fly* calibration for IC-OT-FTMS analysis in negative mode. The concentration of each organic acid was 12.5 nM. For the evaluation of *on-the-fly* calibration for UPLC-OT-FTMS analysis in positive mode, a mixture of 22 unlabeled amino acids and a mixture of four unlabeled peptides (Tables S2 and S3) with a final concentration of each amino acid and peptide was 50.0 nM. For comparing the performance of *on-the-fly* mass accuracy technique at different mass resolutions, pesticide mixture standard solutions (38 mixtures at 100 μg/mL and 2 individual standards) were purchased from Agilent Technologies Inc. (North Kingstown, Rhode Island, USA). Working standard solutions and standard calibration solutions (0.05 to 500 ng/mL) of these pesticides were prepared in Milli-Q water and stored at −20 °C in the dark.

### Ion Chromatography Setup

For negative mode analysis, extracted DOM samples and unlabeled organic acids were analyzed on a Thermo Scientific Dionex ICS-5000^+^–Orbitrap Fusion Tribrid Mass Spectrometer (IC-OT-FTMS). The analytes were run in one dimension with a Dionex IonPac AS11-HC column (2000 Å, 4 μm × 2 mm × 250 mm), a Dionex IonPac AG11-HC 4 μm guard column (4 μm, 2 mm × 50 mm), a Dionex AERS 500e Anion Electrolytically Regenerated Suppressor for External Water Mode (2 mm), and potassium hydroxide (KOH) cartridges. The Dionex AERS 500e Anion is an electrolytic suppressor device positioned after the Dionex IonPac AS11-HC column. It functions by substituting the K^+^ ions from the KOH eluate with H^+^(H_3_O^+^) ions generated electrolytically. This process neutralizes the OH^–^ ions, converting the eluant back to water with an approximate pH of 7. The total analysis run was 20 min with 1 min of re-equilibration, 0.4 mL/min flow, 40 μL of injection volume, and the following gradient: Started with 1 mM KOH, increased to 4 mM KOH 0.1–5.0 min, ramped to 60 mM KOH 5.0–11.0 min, held at 60 mM KOH from 11.0 to 16.0 min, and decreased to 1 mM KOH 16.0–16.1 min. The temperature in the DC compartment was set at 35.0 °C. The H-ESI was set at 3100 V for the negative spray voltage with an ion transfer tube temperature at 350 °C and a vaporization temperature at 300 °C. The three gases on the H-ESI were 50 for sheath gas, 20 for aux gas, and 2 for sweep gas. The Orbitrap was run at a resolution of 500,000 (FWHM at *m*/*z* 200) and a mass range of 85–700 *m*/*z* with an RF lens at 40%. Following the full scan, two MS^2^ were scanned with the ion trap via two filters, dynamic exclusion (*n* = 3 for 60 s) and intensity threshold (min = 1000, max = 1.0e20). Both MS^2^ scans were isolated with the Quadrupole (0.7 *m*/*z*) in the data-dependent acquisition (DDA) approach; however, one fragmentation scan was generated through CID with assisted energy collision and the other fragmentation scan was generated through HCD with stepped energy collision. MS^2^ scan with CID had an automatic gain control (AGC) set at 3.0e4 and a maximum injection time of 50 ms, and the MS^2^ scan with HCD had an AGC of 1.0e4 and a maximum injection time of 50 ms.

Labeled Hippuric acid (ring-^13^C_6_, 99%, Cambridge Isotope) was used as the internal locking mass standard, while labeled α-ketoisovaleric acid, sodium salt (^13^C_5_, 98%, Cambridge Isotope) was used for evaluating the mass locking during the entire retention time. The internal standards for the *on-the-fly* calibration were added in a solution of 96.7% CH_3_CN, 3% H_2_O, and 0.3% NH_4_OH bottle. The locking solution was introduced to the sample via a T-shaped connection after the column separation and before the H-ESI ion source using a Dionex AXP-MS metering pump at a flow rate of 0.200 mL/min (Figure S2). Compound Discoverer 3.2 (Thermo Scientific) was used to identify the DOM compounds, and Skyline Software (MacCoss Lab, University of Washington) was used for organic acid standards detection (see the SI for details). The selected standard mass-labeled hippuric acid was chosen for the following reasons: (1) the signal intensity was consistently at 10^4^ to 10^5^; (2) the standards were stable over a long period in acetonitrile (2 months); (3) the monoisotopic mass is within the studied *m*/*z* range; and (4) the chemical structure is similar to expected analytes.^38,39^

### Liquid Chromatography Setup

For positive mode analysis, DOM-extracted samples, amino acids, and peptide standards were analyzed by a Vanquish Ultra Pressure Liquid Chromatography–Orbitrap Fusion Tribrid Mass Spectrometer (UPLC-OT-FTMS). The analytes were separated on the 1.7 μm ACQUITY UPLC BEH C_18_ reversed-phase column by Waters (130 Å, 1.7 μm, 2.1 mm × 150 mm) (Figure S3). Eluent A, Milli-Q with 0.1% (v/v) formic acid, and eluent B, acetonitrile with 0.1% (v/v) formic acid, were mixed with curve 5 to a flow rate of 0.200 mL/min. The total run lasted 31 min with 7 min re-equilibration and the following gradient: 0–2 min hold at 5% B, ramp to 65% B for 18 min, and then ramp to 100% B for 1 min, and hold at 100% B for 3 min. The H-ESI setting was 3500 V for the positive spray voltage with an ion transfer tube temperature at 300 °C and a vaporization temperature at 225 °C. The three gases on the H-ESI were 35 for sheath gas, 7 for aux gas, and 0 for sweep gas. The OT-FTMS was set similarly to IC-OT-FTMS but was set in positive mode.

Labeled proline-^13^C_5_,^15^N (Sigma-Aldrich) was used as the internal locking mass standard, while labeled valine-^13^C_5_,^15^N (Sigma-Aldrich) was used for evaluating the mass locking during the entire retention time. The internal standards for the *on-the-fly* calibration were added in a solution of 96.7% CH_3_CN, 3% H_2_O, and 0.3% HCOOH. The locking solution was introduced to the sample via a T-shaped connection after the column separation and before the H-ESI ion source using a Dionex AXP-MS metering pump at a flow rate of 0.05 mL/min. Compound Discoverer 3.2 (Thermo Scientific) was used to identify the DOM compounds, and Skyline Software (MacCoss Lab, University of Washington) was used for organic acid, amino acid, and peptide standards detection (see the SI for details).

## Results and Discussion

### Evaluation of *on-the-Fly* Calibration

**Figure 1:**
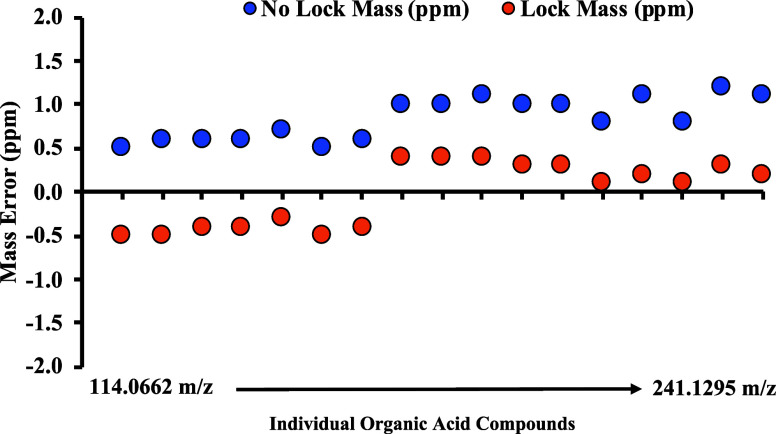
Mass error (ppm) comparison between *on-the-fly* “lock mass” and no “lock mass” of unlabeled organic acid standards analyzed in IC-OT-FTMS in negative mode detection.

To evaluate the *on-the-fly* “lock mass” technique in negative mode, mixtures of 17 unlabeled organic acid standards were analyzed by IC-OT-FTMS with either enabling or disabling user-defined Lock Mass and XCalibur AcquireX. Without the application of “lock mass”, the mass error of these standards ranged from +0.5 to +1.2 ppm and the absolute average mass error of 0.8 ppm ±0.2. However, enabling the “lock mass” by using labeled hippuric acid as an internal standard, the mass error ranged from −0.5 to +0.4 ppm with an absolute average mass error of 0.3 ppm ±0.1 (Figure 1 and Table S1).

In the case of positive mode UPLC-OT-FTMS analysis, the mass error of the 22 unlabeled amino acid and small metabolite standards without “lock mass” ranged from +0.6 to +1.5 ppm and an absolute average of 1.3 ppm ±0.3. However, when the “lock mass” technique was applied using labeled proline, the mass error of the standards ranged from 0 to +0.7 ppm and the absolute average mass error of 0.2 ppm ±0.2 (Figure S4A and Table S2). The *on-the-fly* technique was also tested with four larger unlabeled peptide standards (up to *m*/*z* 574.2330) without “lock mass” mass errors ranging from +0.8 to +1.8 ppm with an average mass error of 1.3 ppm ±0.5. Using the *on-the-fly* “lock mass” technique, the mass error ranged from −0.1 to +0.3 ppm with an absolute average of 0.2 ppm ±0.1 (Figure S4B and Table S3). The 80–800 *m*/*z* range in this study can be confidently calibrated by using only one internal standard. In both UPLC-OT-FTMS (positive mode) and IC-OT-FTMS (negative mode), the *on-the-fly* “lock mass” technique consistently improved the mass accuracy of OT-FTMS to subppm levels, making it comparable to FT-ICR-MS mass accuracy. Another advantage of the *on-the-fly* mass calibration is that it is fully automated, reduces the effect of mass error fluctuations in the entire chromatogram run, and keeps consistent mass accuracy between different samples.

### Evaluating *on-the-Fly* Performance at Different Mass Resolutions

To evaluate the *on-the-fly* mass locking at different mass resolutions, we implemented *on-the-fly* mass locking for 163 pesticides at 10 ng/mL and three different mass resolutions of 60,000, 120,000, and 500,000 (FWHM at *m*/*z* 200). The average mass error for all 163 pesticides at 500,000 FWHM was 0.79 ± 0.2 ppm, while at 120,000 FWHM and 60,000 FWHM, the average mass errors were 0.72 ± 0.27 and 0.55 ± 0.29 ppm, respectively (see Figures S5 and S6). *On-the-fly* mass locking improved the mass accuracy below 1 ppm on average, even at lower mass resolution. The mass error obtained in this study is acceptable according to guidelines used to identify pesticides and chemical substances in food, feed, or veterinary medicine.^40,41^ The guidelines set a mass accuracy of ≤5 ppm, and this value can be applied to identify pesticides in water.

One of the advantages of applying *on-the-fly* mass locking at lower mass resolution is increasing the scan speed without sacrificing the mass accuracy. For example, Figure S7 compares the number of data points across a chromatographic peak for atrazine (one of the pesticides) when the mass resolution and scan speed are 60,000 and 0.4 s, 120,000 and 0.7 s, and 500,000 and 2 s, respectively. The number of data points is higher when the cycle time (scan speed) is shorter at 0.4 s and the mass resolution is 60,000. At the highest mass resolution power (500,000) and lower scan speed (2s), there are fewer data points (12 data points). Analyzing at a higher scan speed (by applying *on-the-fly* mass locking) will increase the sensitivity and lower the detection limit of identified compounds at high mass accuracy, which will be ideal for targeted analysis. However, a higher-resolution MS is still needed for highly complex mixtures to resolve coeluting isobaric compounds.

### *On-the-Fly* Calibration during a Sample Run

**Figure 2:**
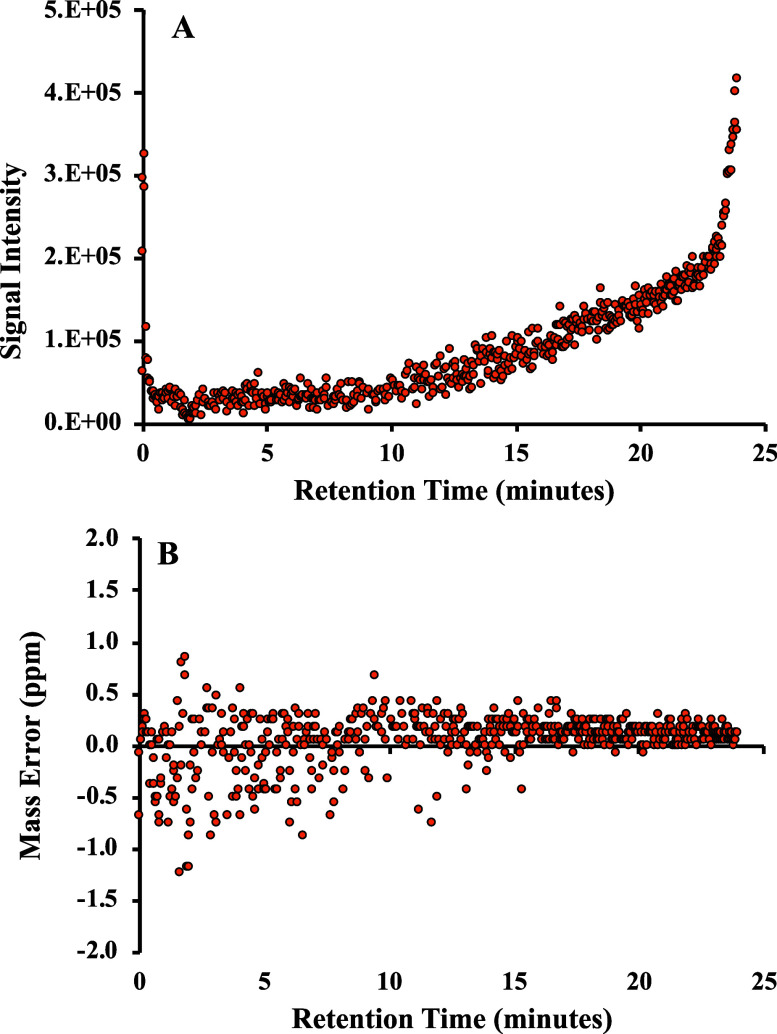
Evaluation of (A) signal intensity and (B) mass error ppm of labeled valine-^13^C_5_,^15^N (124.1001 *m*/*z*) during the entire chromatogram of UPLC-OT-FTMS analysis.

Labeled valine-^13^C_5_,^15^N (120.0568 *m*/*z*) was used to evaluate the *on-the-fly* “lock mass” technique and signal reproducibility during the entire retention time for actual sample analysis in UPLC-OT-FTMS. For example, during the chromatography retention time of the Nueces Bay station NB3 replica R1 sample, the signal intensity of labeled valine ranged from 6.3e3 at 0.1 to 4.1e5 at 24 min (Figure 2A). The intensity mainly was mirror imaged to the gradient of CH_3_CN percentage as it shows low intensity at 95:5 H_2_O:CH_3_CN and increased with increasing CH_3_CN to 100% at 21–24 min. This is attributed to CH_3_CN having a smaller surface tension relative to water, which led to the forming of smaller droplets in the H-ESI and increased the ionization efficiency of ions.^42^ For the mass accuracy, labeled valine showed slightly more scatter accuracy at low signal intensity (low % CH_3_CN) but more consistent accuracy at higher retention time at higher signal intensity. However, the mass error was mainly below 1.0 ppm throughout the sample chromatography run time with an average mass error of 0.2 ppm and a maximum mass error of 1.2 ppm (Figure 2B). It is unfeasible to completely eliminate all drift and fluctuations in the mass accuracy of the Orbitrap, which are impacted by factors such as the stability of the electric field and temperature variations, and achieve zero mass error. Nonetheless, our *on-the-fly* calibration technique substantially mitigates these issues, thereby improving mass accuracy to below 1 ppm.

In the IC-OT-FTMS, we used labeled α-ketoisovaleric acid (120.0568 *m*/*z*) to evaluate the signal reproducibility and mass accuracy of the *on-the-fly* “lock mass” technique during sample analysis in negative mode. In the Nueces Bay station NB3 replica R1 analysis, the signal intensity ranged from 7.2e4 to 1.2e5 throughout the retention time (Figure S8). The trend in the signal intensity was opposite to UPLC as it showed the highest intensity during the first 7 min and then started to decrease. This is attributed to the coeluting of residual chloride and sulfate after 7 min and the formation of carbonate ions as the KOH eluent increases. The presence of salt can lead to ion suppression in H-ESI during these specific retention times and a temporally decreased signal of all compounds. Even though the drop in the intensity is only by a factor of 1.7, it can be improved in the future by removing residual chloride and other inorganic salts during the solid-phase extraction and using the carbonate removal device device after the electronic suppressor.

One of the additional advantages of adopting the *on-the-fly* “lock mass” technique is to provide an independent evaluation of the accuracy and precision of the mass signals during the entire chromatogram and allow additional QC to be monitored within the samples and even between different sample analyses.

### Chromatography Separation

**Figure 3:**
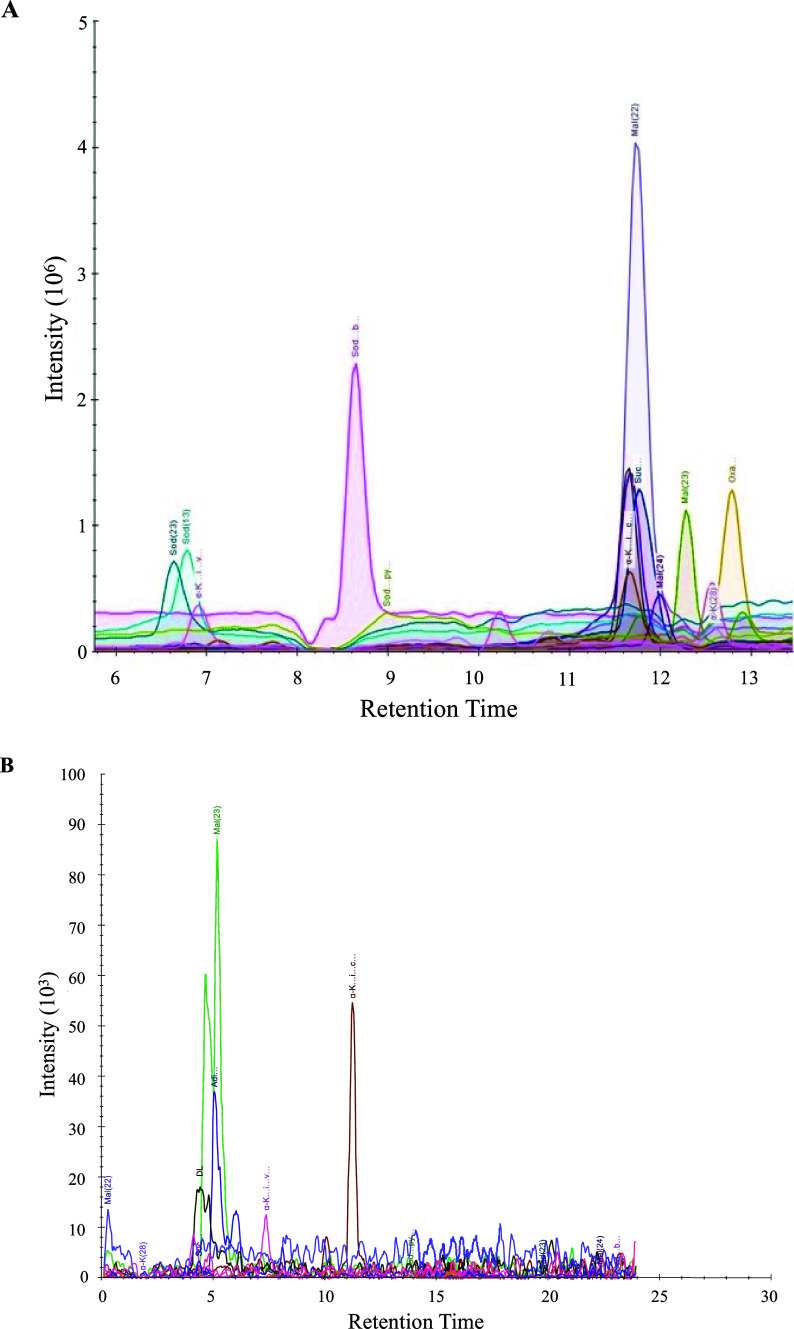
(A) Ion chromatography separation of unlabeled organic acid standards listed in Table S3 in negative mode by IC-OT-FTMS. (B) Liquid chromatography separation of unlabeled organic acid standards listed in Table S3 in negative mode detection using NH_4_OH solution modification of Figure S2.

Evaluating the identification of different small molecules with the two chromatography separation techniques shows that UPLC identified all 22 unlabeled amino acids, small metabolites, and four peptides (Tables S2 and S3). They were separated along a retention time span from 1.78 to 7.75 min at 50.0 nM. The 17 organic acids showed a chromatography separation from 6.45 to 12.8 min at a concentration of 12.5 nM (Table S1 and Figure 3A). These results indicate that IC can separate organic acid standards at low concentrations, just as UPLC is ideal for separating amino acids and peptides.

As a proof of concept of chromatography separation, organic acids were also analyzed by UPLC-OT-FTMS in negative mode. However, we could not identify any 17 organic acids at 12.5 nM. For further testing, HCOOH was replaced in the standard internal bottle with NH_4_OH to enhance the deprotonation of these organic acid standards and reanalyzed them in negative mode. By comparing the UPLC (Figure 3B) to the IC analysis in negative mode, only five organic acids were detected in the UPLC out of the 17 organic acids detected in the IC at the same concentration. Furthermore, the peak integration of the five organic acids was 7 to 59 times higher in IC compared to the UPLC technique. Many standards could not be distinguished from the background, and the few standards with recognizable peaks suffered in quality and intensity compared to IC results. This clearly showed that IC has significantly higher sensitivity in detecting and quantifying these organic acids than the UPLC technique even with negative mode detection and using NH_4_OH to enhance deprotonation. Organic acids are better separated at higher pH due to their acidic functional groups; thus, KOH as a mobile phase in IC provides a better separation at the eluent pH level over a low pH with formic acid in UPLC. Unlike the IC column, the C18 UPLC column (a silica-based column) cannot handle higher pH levels. This can lead to low separation performance, low peak quality, or signal below the detection limit due to low ionization. In addition, there is a weak interaction of the detected organic acid on the C18 UPLC column due to the hydrophobicity of the stationary phase and the hydrophilicity of these organic acids.

Since DOM compounds have various physicochemical characteristics, a single chromatography method will not comprehensively separate and quantify their complex mixture. Although semipolar and positively charged compounds are well separated with the UPLC technique, highly polar and anionic compounds are more efficiently retained with the IC column, as explained in Petucci et al. and Wang et al.^15,43^ A successful chromatography reduces a sudden overload of compounds at a specific retention time, increasing ion efficiency, higher sensitivity, and potentially better fragmentation data.

### Reproducibility of DOM Samples

To evaluate the performance of two different normalization techniques in minimizing the instrumental analysis variability and assess the reproductivity between different DOM samples, principal component analysis (PCA) was applied on replicas of 18 Nueces Bay DOM samples that were processed through two normalization methods: (1) constant sum normalization and (2) SERRF.^37^ The PCA includes replicas of all 18 DOM samples and four QC vial injections after every nine DOM sample injections.

**Figure 4:**
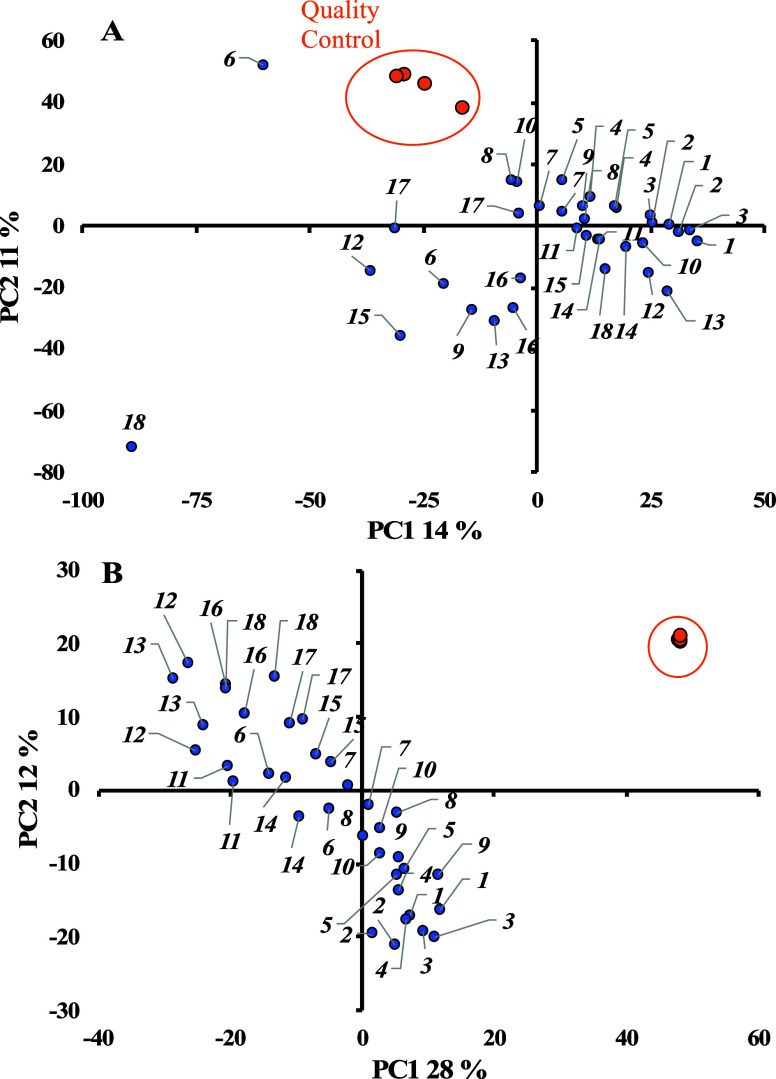
PCA plots of NB samples analyzed by IC-OT-FTMS. (A) Data normalized with constant sum. (B) Data normalized with SERRF. Quality control samples are marked in an orange circle.

For IC-OT-FTMS data, by using constant sum normalization, the first two PCs account for 25% of the differences (Figure 4). QC samples (circled in orange) showed some separation from each other, indicating a significant drift during sample analysis within a day of analysis. Two sample outliers, without their replica, fall in the bottom left and top left quadrants, which could have skewed the overall PCA and interpretations. In the PCA plot with SERRF normalization, the first two PCs explain about 40% of the differences in the samples. QC injections are clustered tightly together, showing improved overall reproducibility.

Additionally, SERRF has clustered the outliers (observed in constant sum normalization) closer to their replica samples, making all stations evenly distributed along the top-left and bottom-right quadrants. When the data was normalized with the constant sum method, the average relative standard deviation of the peak area of QC-detected compounds was 34.0%. In contrast, the data normalized with SERRF were 4.4%. As the same QC sample was re-injected at specific increments, a lower relative standard deviation indicates a reduced variation of instrumental measurement errors of the data.

For UPLC-OT-FTMS data, PCA was plotted for each normalization technique, constant sum normalization, and SERRF in Figure S9. The first two PCs with constant sum normalization explained about 58% of the variability of the samples, and SERRF explained 49% of the variability of the samples. As observed in the PCA of IC-OT-FTMS, there is a clear improvement in the reproducibility of the QC samples (circled in orange) with SERRF compared to constant sum normalization. Although the analysis was conducted within 1 day, the overall reproducibility with SERRF improved drastically compared to constant sum normalization. The calculated average standard deviation of the peak area of QC-detected compounds with the constant sum normalization was 28.1%, whereas the average standard deviation normalized with SERRF was 3.9%. As a reference, the recommended cutoff for intensity reproducibility with FT-ICR-MS is 10%.^44^

These results clearly show the need for normalization to minimize the instrumental analysis variability. Evaluating the reproductivity is essential for analyzing a complex mixture like DOM to highlight the differences between chemical characterization within or between different aquatic ecosystems, whereas the SERRF method using quality control pool samples showed a better removal of systematic variation and produced a more robust ecological and biogeochemical interpretation of the DOM chemical composition changes within or between different aquatic ecosystems.

### DOM Molecular-Level Characterization

**Figure 5:**
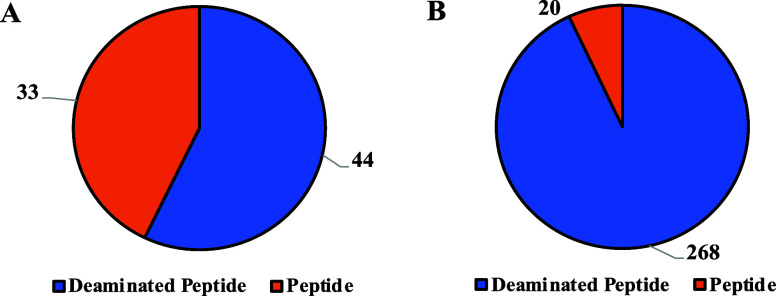
Pie charts of numbers of peptides and deaminated peptides putatively structurally elucidated in (A) UPLC-OT-FTMS in positive mode and (B) IC-OT-FTMS in negative mode.

With IC-OT-FTMS in negative mode, a total of 1432 compounds were detected, with 338 matching the formulas of peptides and deaminated peptides in-house structural database^45^ and 197 compounds matching the formula of our in-house organic acids structural database. Out of the 338 peptides and deaminated peptide matches, 298 (88%) had MS^2^ fragmentation spectra, and out of 197 organic acids, 188 (96%) had MS^2^ fragmentation spectra. We used the mzCloud mass spectral database (Thermo Fisher) and our in-house experimental mzVault of chemical standard organic acid fragmentation database. Additionally, we applied *in silico* fragmentation (Mass Frontier software, Thermo Fisher) coupled with mzLogic (Thermo Fisher) to putatively elucidate the molecular structures of the peptides and deaminated peptides that are not present in the spectral libraries. To rank the different structures, we used a FiSh score, calculated by comparing the experimental fragments with *in silico* fragmentation. The higher the score, the more fragments matched the suggested structure versus unmatched fragments (with a cutoff of 70% FiSh score). Additionally, in UPLC-OT-FTMS positive mode, a total of 915 compounds were detected, with 85 matching the peptide formula and deaminated peptides in the in-house structural database. Out of the 85 matches, 80 (94%) had MS^2^ fragmentation spectra and a total of 77 peptides and deaminated peptides were putatively structurally elucidated using *in silico* fragmentation. In this study, we putative structurally elucidate a total of 53 peptides and 312 deaminated peptides using both modes. Of the 53 peptides, 33 were detected in UPLC and 20 in the IC technique. Of 312 deaminated peptides, 268 were detected in IC and only 44 were detected in the UPLC technique (Figure 5).

Due to their physiochemical characteristics, it is expected to detect more peptides with UPLC (positive mode) and more deaminated peptides with IC (negative mode). See Text S4 in the Supporting Information for more explanation. Since some peptides have multiple terminal amine groups, stepwise deamination processes could generate deaminated peptides with one or more remaining terminal amine groups. These incomplete deaminated peptides could potentially be ionized in both modes. Examining our Nueces Bay DOM samples, only five compounds were detected in both ionization modes (one peptide and four deaminated peptides). For example, peptide PP_169 has one carboxylic group, which can be deprotonated at high pH and carry a negative charge, and two amine functional groups with high p*K*_a_ (>10) and can carry a positive charge at low pH. The deaminated peptide DP_1_323 underwent a single deamination of the precursor peptides but still has another terminal amine group in addition to the carboxylic acid functional group, which can potentially be ionized in both positive and negative modes depending on eluent pH (Figure S10).

**Figure 6:**
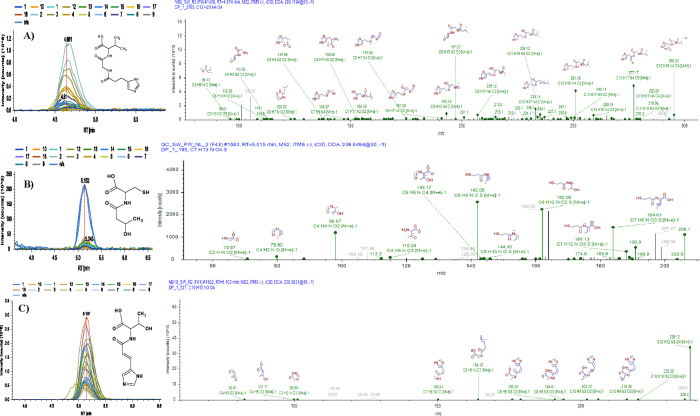
Examples of some putative structure of deaminated peptides detected in Nueces Bay DOM samples with their MS^**2**^ fragmentation spectra and chromatogram peaks. (A) DP_1_2785: reductive deamination of Val-Gly-His with chemical formula C_13_H_20_N_4_O_4_. (B) Deaminated peptide DP_1_527: reductive deamination of Cys-Thr with chemical formula C_7_H_13_NO_4_S. (C) Deaminated peptide DP_1_195: histidine deamination of His-Thr with chemical formula C_10_H_13_N_3_O_4_.

All other 283 in IC and 72 in UPLC did not overlap and were uniquely detected in their corresponding detection modes. For example, Figure 6 demonstrates a putative structure, chromatogram, and the MS^2^ spectrum of three deaminated peptides identified in Nueces Bay surface water samples IC in negative mode. The first compound was a single reductive deamination of the Val-Gly-His peptide (Figure 6A). The second compound is a single reductive deamination of the Cys-Thr peptide (Figure 6B). The third compound is a single-histidine deamination of the His-Thr peptide (Figure 6C). Examples of detected peptides found in Nueces Bay surface water samples that were detected by UPLC in positive mode were Lys-Lys-Pro, Val-Val-Lys-Ser, and Pro-Lys-Ala-Ser (Figure S11). These results highlight the need for combining these two techniques to have a more comprehensive method for DOM characterization. The application of the combined IC and UPLC techniques is not limited to DOM chemical characterization. It can be applied to analyze other complex compound mixtures like metabolites, detecting anthropogenic pollutants like pesticides and endocrine-disrupting chemicals in natural and biological samples.

In the marine environment, proteins are either hydrolyzed to peptides and free amino acids by microorganisms for metabolic energy followed by CO_2_ remineralization or hydrolyzed to peptides and then deaminated by anaerobic bacteria to produce energy. The overall low number of small peptides detected in Nueces Bay’s DOM samples can be attributed to being labile compounds for microbial biochemical assimilation and catabolism processes. However, some peptides could not be completely hydrolyzed so that microbes can be deaminated under fermentation conditions to deaminated form.^45^ The relatively high number of deaminated peptides in the surface water is due to the continuous deamination process (i.e., by microbes) over time and indicates their relative refractory nature compared to the peptides. The deaminated structure, as it still maintains the carbon skeleton or the precursor peptide, can be used to trace the source, microbial communities, and enzymes, ultimately fingerprinting a peptide’s bioavailability and transformation within the marine environment.

## Conclusions

To identify, elucidate, and quantify DOM, high-resolution and high mass accuracy, along with an enhanced separation via retention time to reduce overall complexity, high coverage of fragmentation MS^2^ data, and high reproducibility are required. This method provides a solution for all four requirements for the most comprehensive surface water DOM analysis. The *on-the-fly* internal mass calibration achieves high mass accuracy, producing a consistent subppm error with UPLC and IC without high analytical expenses. The proposed setup for “lock mass” is versatile and can be applied to any high-resolution mass spectrometer, and standards can be chosen according to the study’s needs. The introduction of IC improves the retention and separation of highly polar and anionic compounds with low background noise compared to previous methods such as HILIC, making it an ideal complementing technique for negative mode detection combined with UPLC in positive mode detection. Thus, we recommend introducing an IC system to broaden the detection coverage with a mass spectrometer, especially when working with complex mixtures containing various physicochemical compounds. SERRF, a random forest-based data normalization, enhanced reproducibility to below 5% for UPLC and IC, reducing overall systematic error and biased interpretation compared to traditional processes such as a constant sum for this DOM sample set.

## Supporting Information Available

Additional details on DOM extraction procedure data and processing of mass spectra. It also includes Instrumental schematic diagrams of both IC-OT-FTMS and UPLC-OT-FTMS systems, supplemental figures, and tables. The Supporting Information is available free of charge at https://pubs.acs.org/doi/10.1021/acs.analchem.3c02599.

Solid-phase extraction; compound discoverer for ion chromatography; compound discoverer for liquid chromatography; chemical functional groups and ionization efficiency; sampling map; instrumental schematic of IC-OT-FTMS; instrumental schematic of UPLC-OT-FTMS; mass error (ppm) comparison between “lock mass” and no “lock mass” of unlabeled amino acid, small metabolite standards, and unlabeled peptides; mass error (ppm) for a mixture of 163 targeted pesticides at different MS resolutions; average mass error (ppm) for a mixture of 163 targeted pesticides at different MS resolutions; comparison of the number of scans (data points) per chromatographic peak of atrazine obtained at three different mass resolutions and scan speeds; evaluation of the signal intensity of labeled α-ketoisovaleric acid during the entire chromatogram of IC-OT-FTMS analysis; PCA plots of NB samples analyzed by UPLC-OT-FTMS data normalized with constant sum and with SERRF; tentative structure of a deaminated peptide and a peptide that were detected in both positive and negative mode; UPLC-OT-FTMS chromatographic peaks and FiSh fragmentation spectra of three detected peptides; a list of unlabeled organic acid standards and their mass error results with and without “lock mass”; a list of unlabeled amino acid and small metabolite standards and their mass error results with and without “lock mass”; and a list of unlabeled peptide standards and their mass error results with and without “lock mass” (PDF)
