# Supplementary material for: Toward a More Comprehensive Approach for Dissolved Organic Matter Chemical Characterization Using an Orbitrap Fusion Tribrid Mass Spectrometer Coupled with Ion and Liquid Chromatography Techniques
Source: Anal Chem. 2024 Feb 19;96(9):3744–53. doi: 10.1021/acs.analchem.3c02599 (PMC10918622; doi:10.1021/acs.analchem.3c02599)
Supplement: Supplementary file 1 — ac3c02599_si_001.pdf [file ac3c02599_si_001.pdf]

Supplement Information for

Toward More Comprehensive Approach for Dissolved Organic Matter Chemical Characterization Using Orbitrap Fusion Tribrid Mass Spectrometer Coupled with Ion and Liquid Chromatography Techniques

Daniela Bergmann[1]* Jessie Matarrita-Rodríguez[1,3] & Hussain Abdulla[1,2]*

[1] Department of Physical and Environmental Sciences, Texas A&M University – Corpus Christi, TX, USA

[2] Center for Water Supply Studies, Texas A&M University – Corpus Christi, TX, USA

[3] Centro de Investigación en Contaminación Ambiental (CICA), Universidad de Costa Rica, San José Costa Rica

*Corresponding authors danielabergmann1@web.de, hussain.abdulla@tamucc.edu

Table of Contents

Text S1. Solid-Phase-Extraction………………………………………………………… S3

Text S2. Compound Discoverer for Ion Chromatography………………………………. S3 -S4

Text S3. Compound Discoverer for Liquid Chromatography…………………………... S4- S5

Text S4. Chemical functional groups and Ionization efficiency………………………… S5

Figure S1. Sampling map……………………………………………………………….. S6

Figure S2. Instrumental schematic of IC-OT-FTMS……………………………………. S6

Figure S3. Instrumental schematic of UPLC-OT-FTMS……………………………….. S7

Figure S4. Mass error (ppm) comparison between "lock mass" and no "lock mass" of unlabeled amino acid, small metabolite standards and unlabeled peptides……………... S8

Figure S5. Mass error (ppm) for a mixture of 163 targeted pesticides at different MS resolutions……………………………………………………………………………. S9

Figure S6. Average mass error (ppm) for a mixture of 163 targeted pesticides at different MS resolutions……………………………………………………………………. S10

Figure S7. Comparison of the number of scans (data points) per chromatographic peak of Atrazine obtained at 3 different mass resolutions and scan speeds…………………... S10

Figure S8. Evaluation of the signal intensity of labeled α-ketoisovaleric acid during the entire chromatogram of IC-OT-FTMS analysis………………………………………. S11

Figure S9. PCA plots of NB samples analyzed by UPLC-OT-FTMS data normalized with constant sum and with SERRF…………………………………………………... S12

Figure S10. Tentative structure of a deaminated peptide and a peptide that were detected in both positive and negative mode……………………………………………. S13

Figure S11. UPLC- OT-FTMS Chromatographic peaks and FiSh fragmentation spectra of three detected peptides………………………………………………………………. S13- S14

Table S1. A list of unlabeled organic acid standards and their mass error results with and without "lock mass"……………………………………………………………… S15

Table S2. A list of unlabeled amino acid and small metabolite standards and their mass error results with and without "lock mass"……………………………………………… S16

Table S3. A list of unlabeled peptide standards and their mass error results with and without "lock mass"……………………………………………………………………... S17

References Cited S17

Text S1.

Solid-Phase-Extraction. In brief, PPL cartridge was activated with two cartridge volumes (2x 6mL) of LC/MS grade methanol followed by one cartridge volume of Milli-Q water. Then, the acidified surface water sample was passed through the PPL cartridge, followed by washing with three cartridge volumes of formic acid solution (pH 2) for more efficient salt removal. The extracted DOM was eluted with two cartridge volumes of Optima LC/MS grade methanol (Thermo Scientific). Two Milli-Q samples of 1000mL were processed with the same SPE extraction procedure and used as a method-blank to account for background contamination. The DOM sample in methanol was then dried in a CentriVap benchtop vacuum concentrator and redissolved with 10mL of Milli-Q water due to high recovery of DOM compounds.

The pool QC contained surface water, porewater and nepheloid layer samples, but they were not discussed in this study due to a large number of samples.

Text S2.

Compound Discoverer for Ion Chromatography. Compound Discoverer 3.2 (Thermo Scientific) was used to identify the DOM compounds. The adaptive curve was used with a 2 min maximum shift and 5ppm mass tolerance to align the retention times of chromatography spectra. For compound identification, the following four criteria were met: 1) signal-to-noise threshold of 3 2) minimum of 8 scans per peak 3) minimum of one isotope with 4) peak intensity of 50,000. However, the adducts were changed to negative charged compounds: $[2M + FA - H]^{-1}$, $[2M - H]^{-1}$, $[2M - H + HAs]^{-1}$, $[M - Cl]^{-1}$, $[M + FA - H]^{-1}$, $[M - 2 H + K]^{-1}$, $[M - H]^{-1}$, $[M – H + HAs]^{-1}$, $[M – H - TFA]^{-1}$, $[M – H - H_2O]^{-1}$. The maximum element combination was set to C_{90} H_{190} N_{10} O_{30} P_1 S_2. The role used to constrain the elemental compositions were explained in our previously published papers[1-4]. In brief, we applied a modified version of the rules set in Kind

and Fiehn[5], which requires that formulas satisfy the following inequalities: $H/C < 2.50$, $O/C \leq 1.20$, $O/P \geq 3.00$, and $N/C < 0.50$. All assigned formulas were further tested for the physical existence of chemical structures using LEWIS and SENIOR chemical rules, again according to Kind and Fiehn[5]. The molecular ^{13}C isotope and ^{34}S isotope peaks (when they were detected above the S/N threshold) were also validated with the chemical building block approach (e.g., CH_2 homologies series) described by Koch et al.[6] .The detected compounds were then compared to mzCloud, Predict Compounds, m/z Logic, and a Search Mass List (Peptides, Deaminated Peptides and organic acids). The calculated peak areas were normalized with "Constant Sum" when SERRF was not applied. When SERRF was applied, the "Normalized Area" was replaced with "Apply SERRF QC Correction (beta)" with the following criteria: 1) minimum QC coverage 50% 2) maximum QC area RSD 30% 3) maximum corrected QC Area RSD 25% with one batch (indicating that the same QC was used for all QC vials) and 500 trees.

Text S3.

Compound Discoverer for Liquid Chromatography. Compound Discoverer 3.2 (Thermo Scientific) was used to identify the DOM compounds. The adaptive curve was used with a 2 min maximum shift and 5ppm mass tolerance to align the retention times of chromatography spectra. For compound identification, the following four criteria were met: 1) signal-to-noise threshold of 3 2) minimum of 8 scans per peak 3) minimum of one isotope with 4) peak intensity of 50,000.

For the ion detection, the following adducts were considered: $[M + H]^{+1}$, $[M + K]^{+1}$, $[2M + H]^{+1}$, $[M + Na]^{+1}$, $[M + NH_4]^{+1}$, $[M + ACN + H]^{+1}$, $[M + ACN + Na]^{+1}$, $[M + DMSO + H]^{+1}$, $[M + H + MeOH]^{+1}$, $[M + H\text{-}H_2O]^{+1}$, $[M + H - NH_3]^{+1}$, $[2M + ACN + H]^{+1}$, $[2M + ACN + Na]^{+1}$, $[2M + FA + H]^{+1}$, $[2M + K]^{+1}$, $[2 M + Na]^{+1}$, [2M +

$NH_4]^{+1}$, $[M + 2H]^{+2}$, $[M + ACN + 2H]^{+2}$, $[M + H + K]^{+2}$, $[M + H + Na]^{+2}$, $[M + H + NH_4]^{+2}$, $[M + 3H]^{+3}$.

The maximum element combination was set to $C_{90} H_{190} N_{10} O_{30} P_1 S_2$. The roles used to constrain the elemental compositions were similar to what we used for IC-OT-FTMS. The detected masses were then compared to mzCloud, Predict Compounds, m/z Logic, and a Search Mass List (Peptides and Deaminated Peptides). The calculated peak areas were normalized with "Constant Sum" when SERRF was not applied. When SERRF was applied, the "Normalized Area" was replaced with "Apply SERRF QC Correction (beta)" with the following criteria: 1) minimum QC coverage 50% 2) maximum QC area RSD 30% 3) maximum corrected QC Area RSD 25% with one batch (indicating that the same QC was used for all QC vials) and 500 trees.

Text S4 Chemical functional groups and Ionization efficiency

In general, the presence of a terminal amine functional group in the peptide enhances the ionization efficiency at low eluent pH through protonation to ammonium ion. However, removing the terminal amine groups from the peptide through microbial deamination processes[40], generates a deaminated peptide that is likely to ionize through deprotonation of the remaining terminal carboxyl group at higher eluent pH, which explains the more significant number of deaminated peptides detection with IC-OT-FTMS in negative mode.

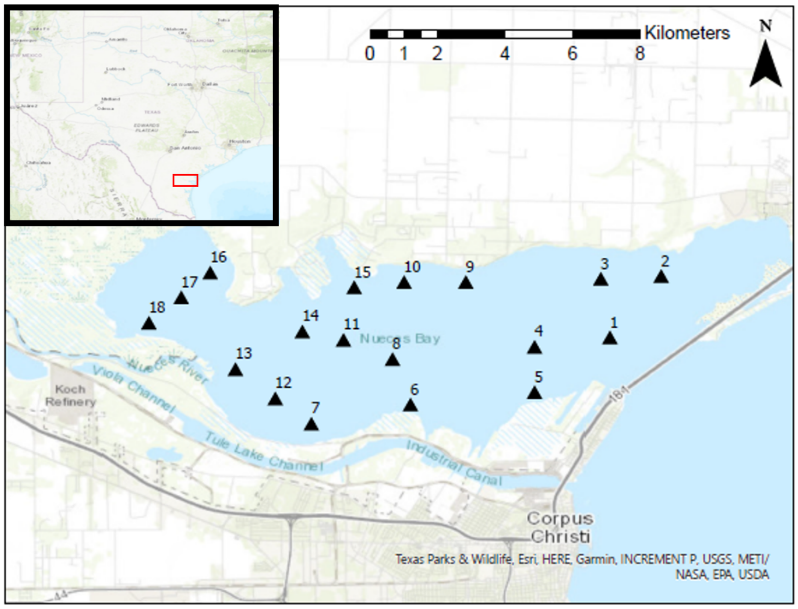

Figure S1. Sampling map of Nueces Bay, Texas, depicting 18 sampling locations.

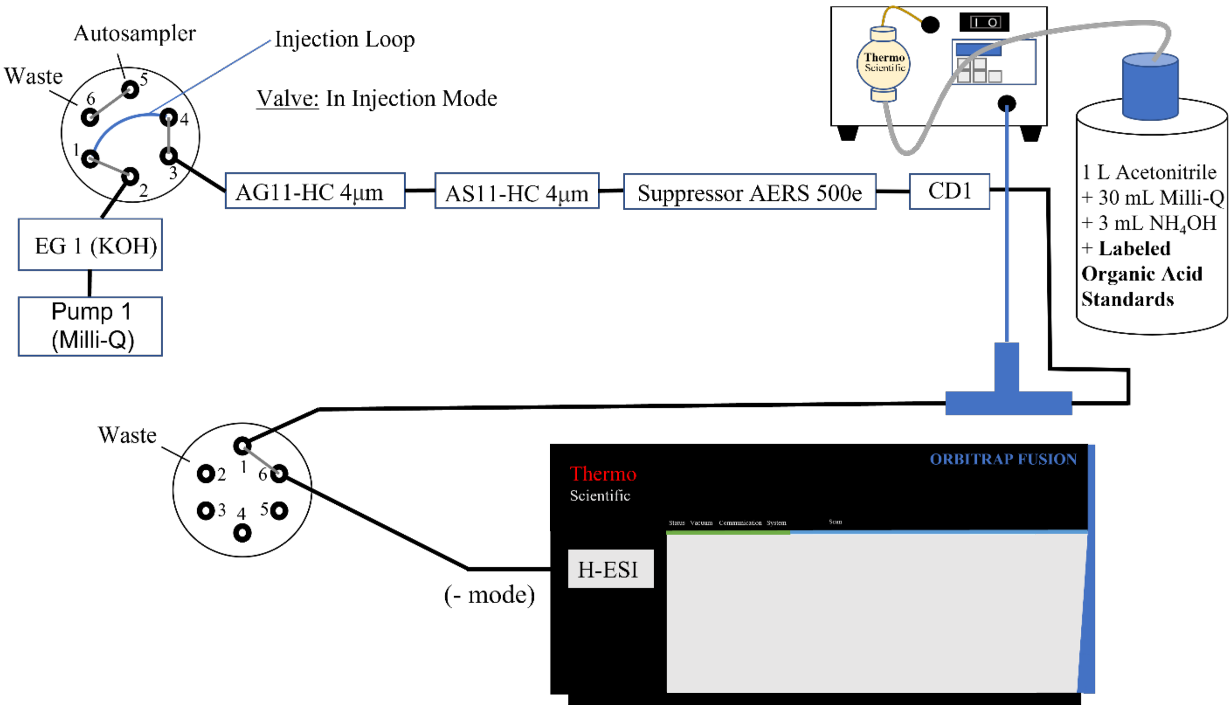

Figure S2. Instrumental schematic of ion chromatography attached to Orbitrap Fusion Tribrid Mass Spectrometer in negative mode detection.

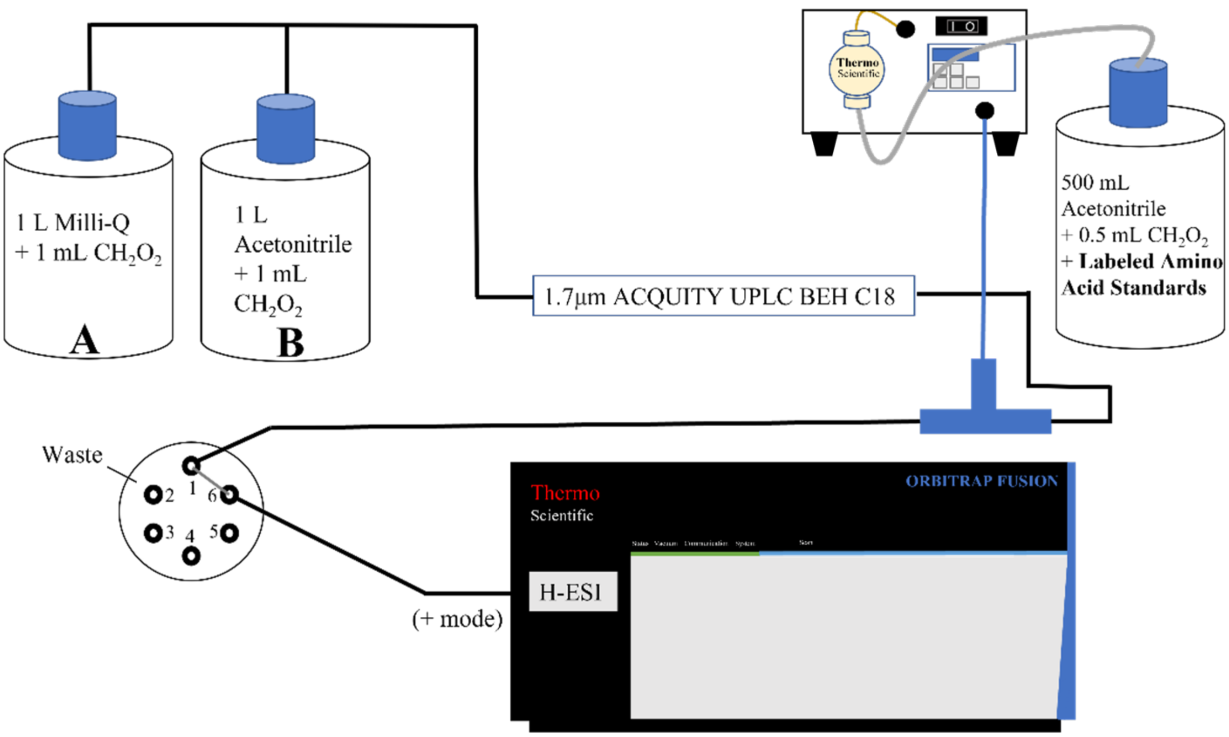

Figure S3. Instrumental schematic of Ultra performance liquid chromatography attached to Orbitrap Fusion Tribrid Mass Spectrometer in positive mode detection.

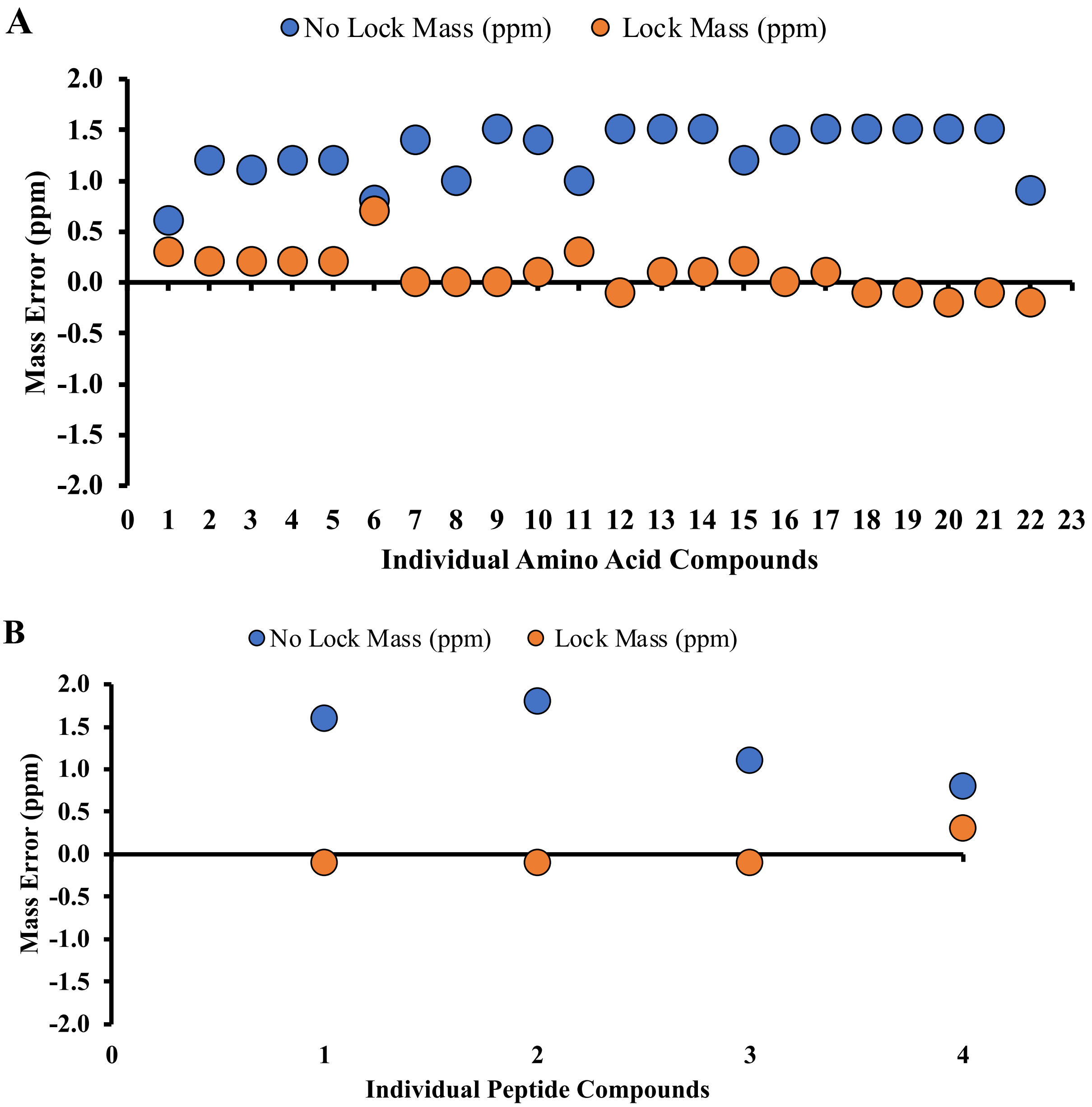

Figure S4. Mass error (ppm) comparison between "lock mass" and no "lock mass" of **A)** unlabeled amino acid and small metabolite standards **B)** and unlabeled peptides with liquid chromatography in positive mode detection.

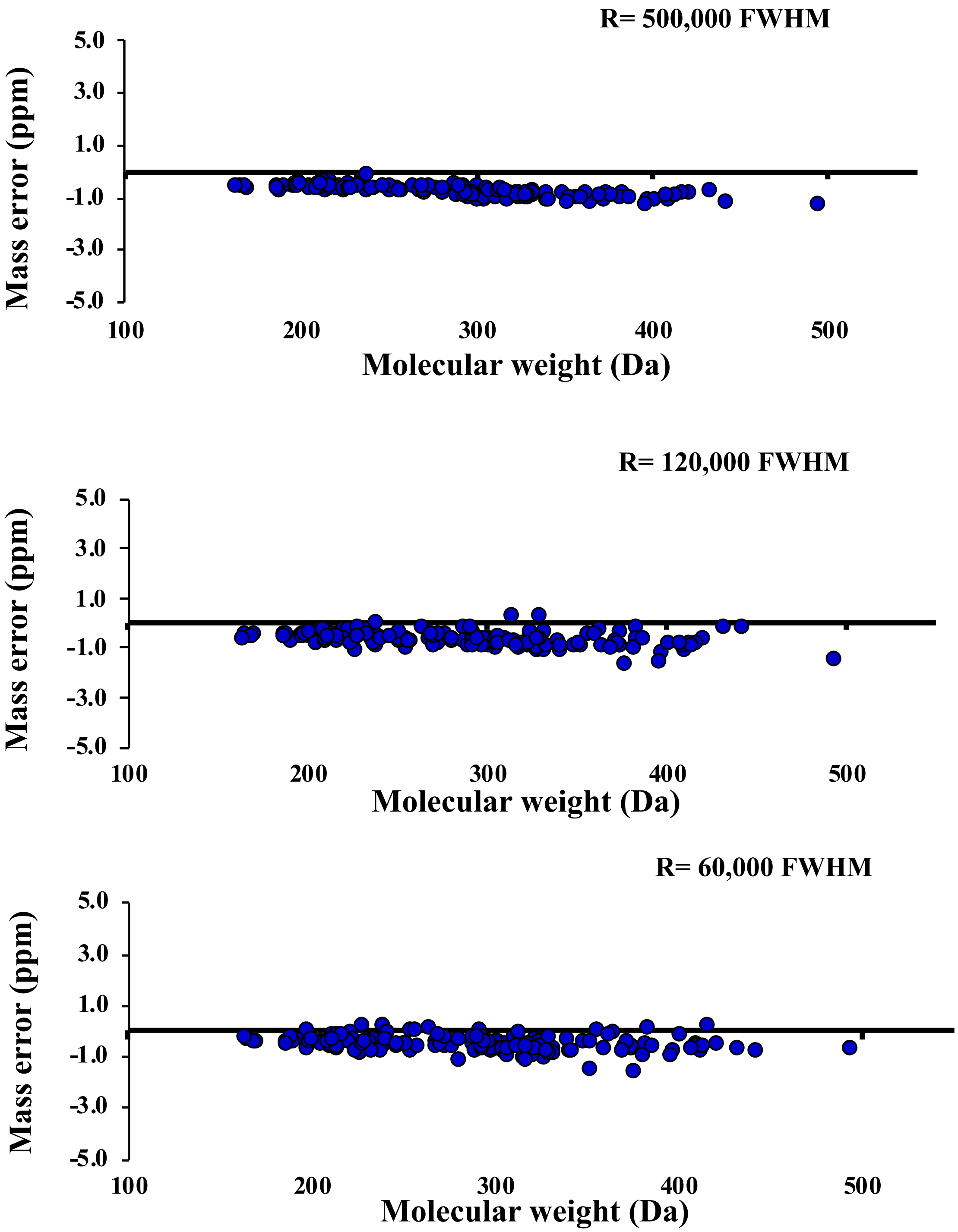

Figure S5. Mass error (ppm) for a mixture of 163 targeted pesticides (10 ng/mL) at different MS resolutions (500,000, 120,000 and 60,000) using UPLC-OT-FTMS in positive mode with *on-the-fly* mass locking.

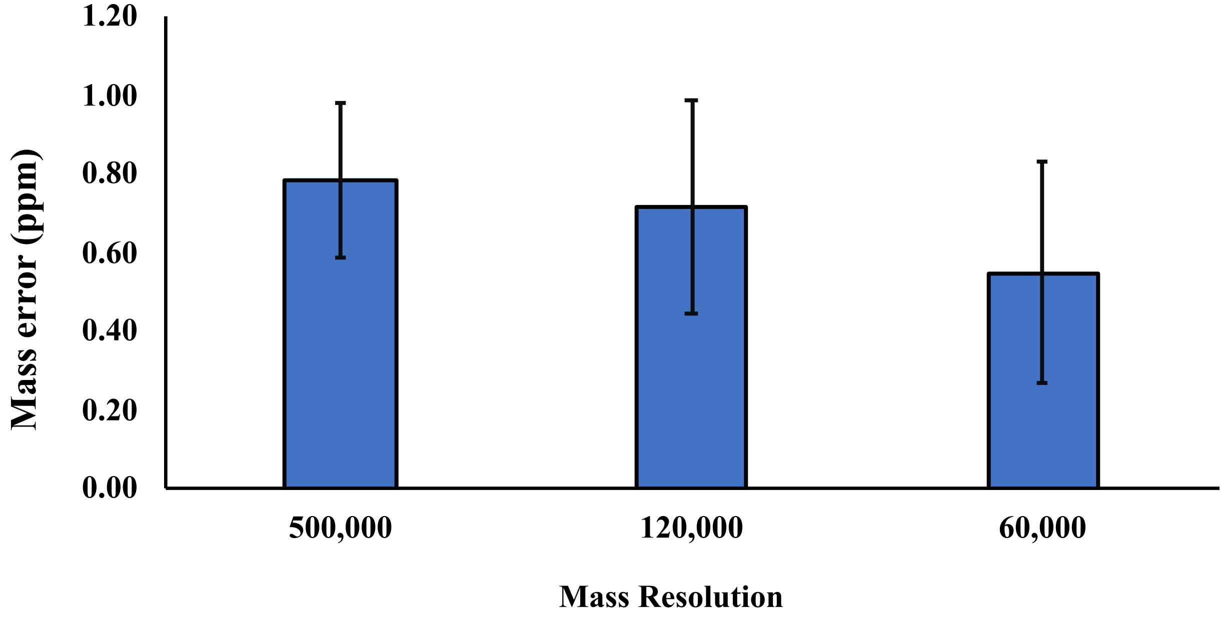

Figure S6. Average mass error (ppm) for a mixture of 163 targeted pesticides (10 ng/mL) at different MS resolution (500,000, 120,000 and 60,000) using UPLC-OT-FTMS in positive mode with *on-the-fly* mass locking.

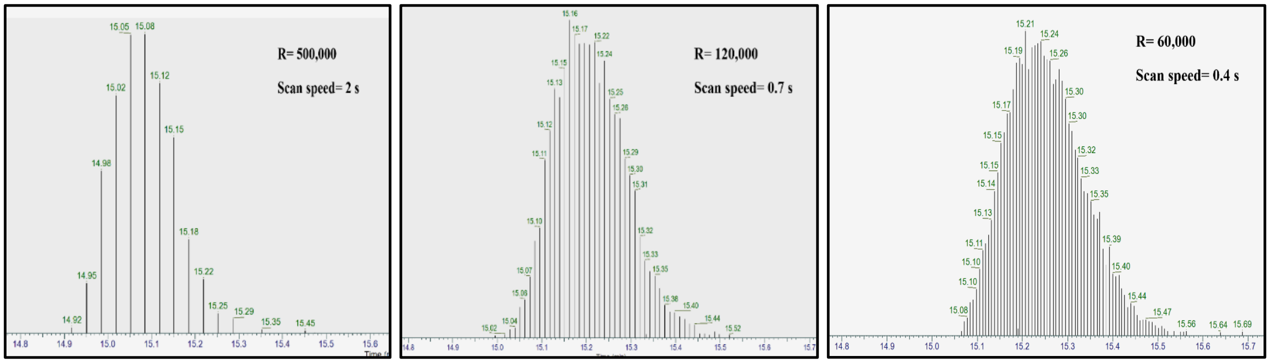

Figure S7. Comparison of the number of scans (data points) per chromatographic peak of Atrazine obtained at 3 different mass resolutions and scan speeds.

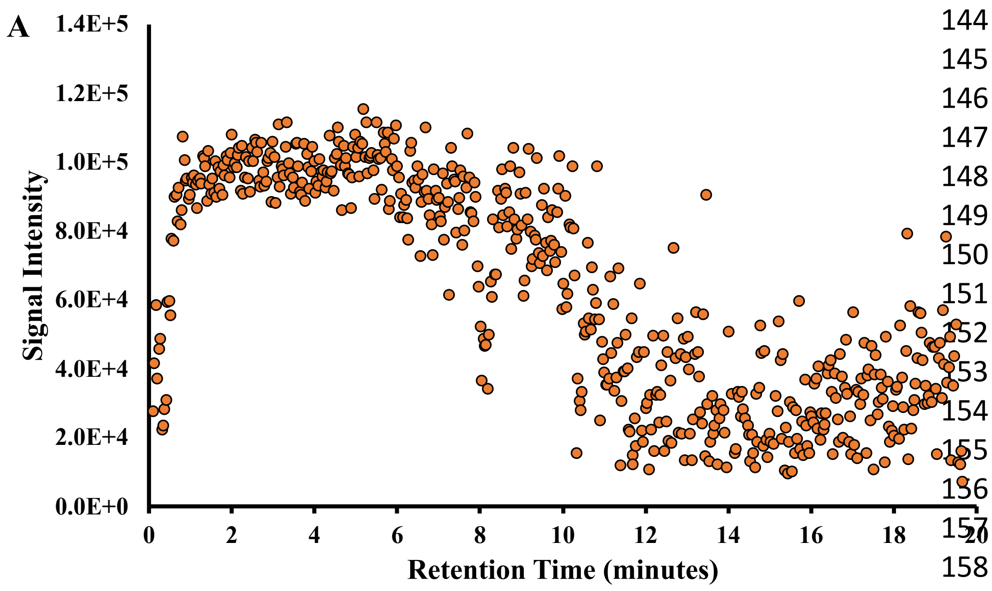

Figure S8. Evaluation of the signal intensity of labeled α-ketoisovaleric acid (120.0568 m/z) during the entire chromatogram of IC-OT-FTMS analysis.

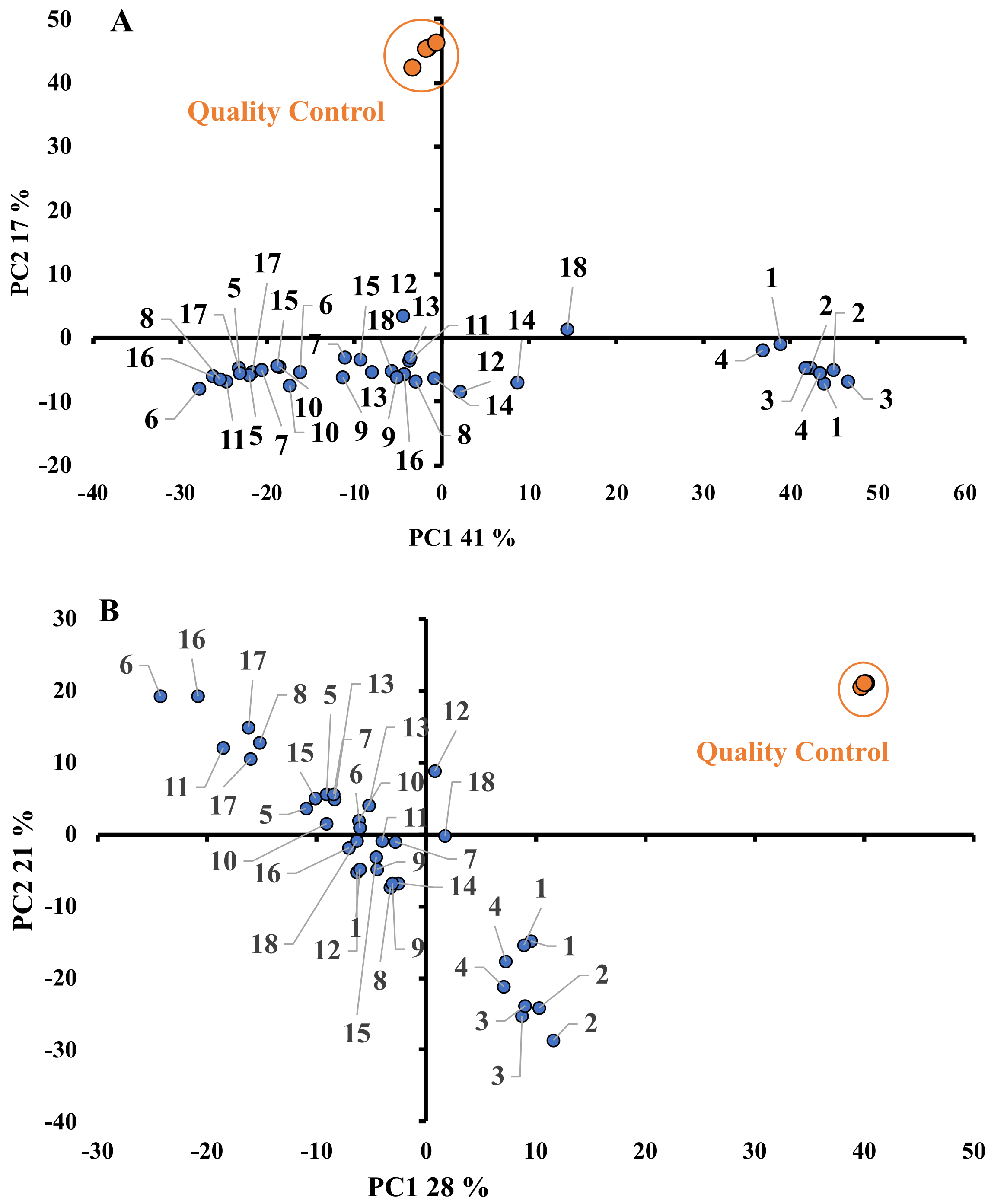

Figure S9. PCA plots of NB samples analyzed by UPLC-OT-FTMS **A**) data normalized with constant sum **B)** data normalized with SERRF. Quality control samples are marked in an orange circle.

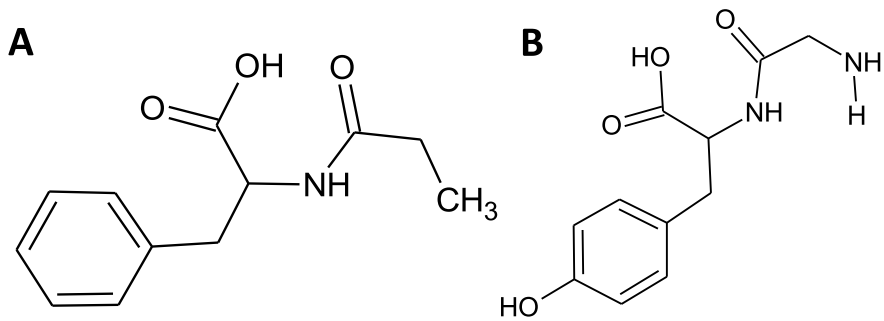

Figure S10. Tentative structure of **A)** deaminated peptide DP_1_323 and **B)** peptide PP_169 that were detected in both positive and negative mode.

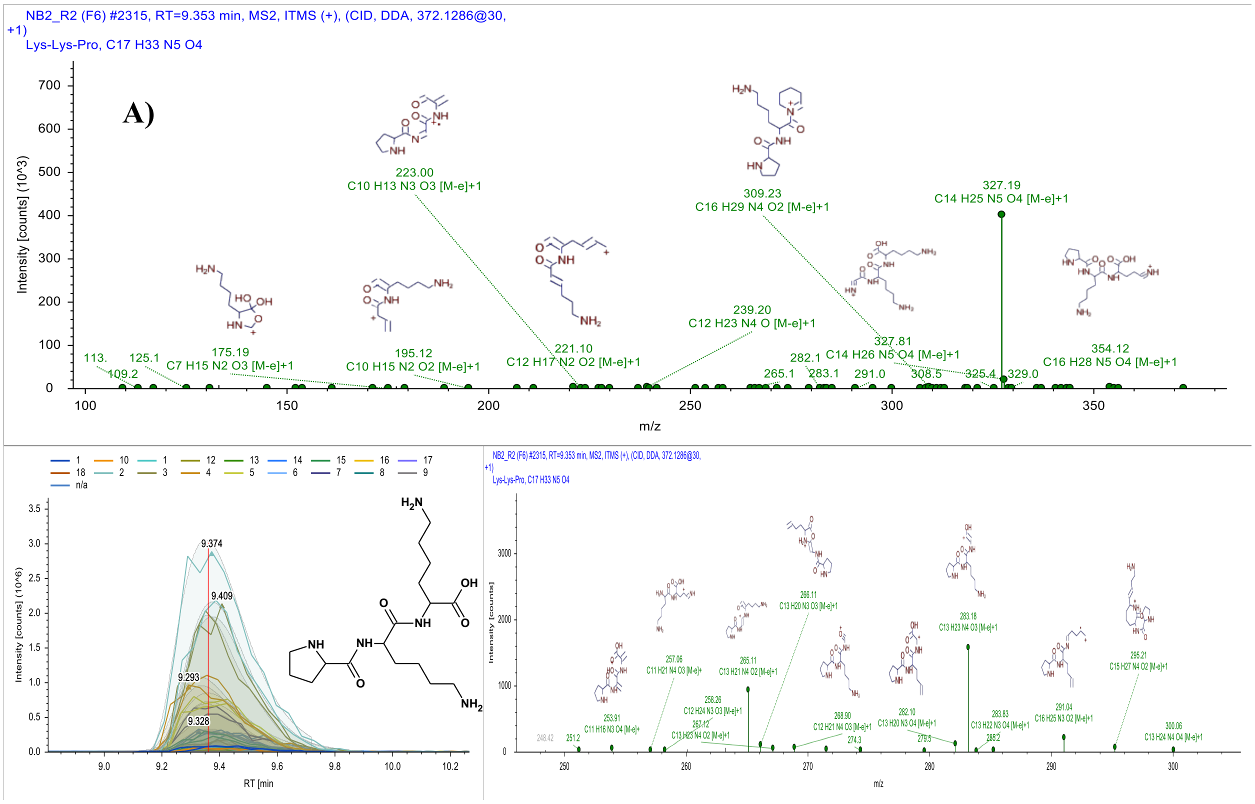

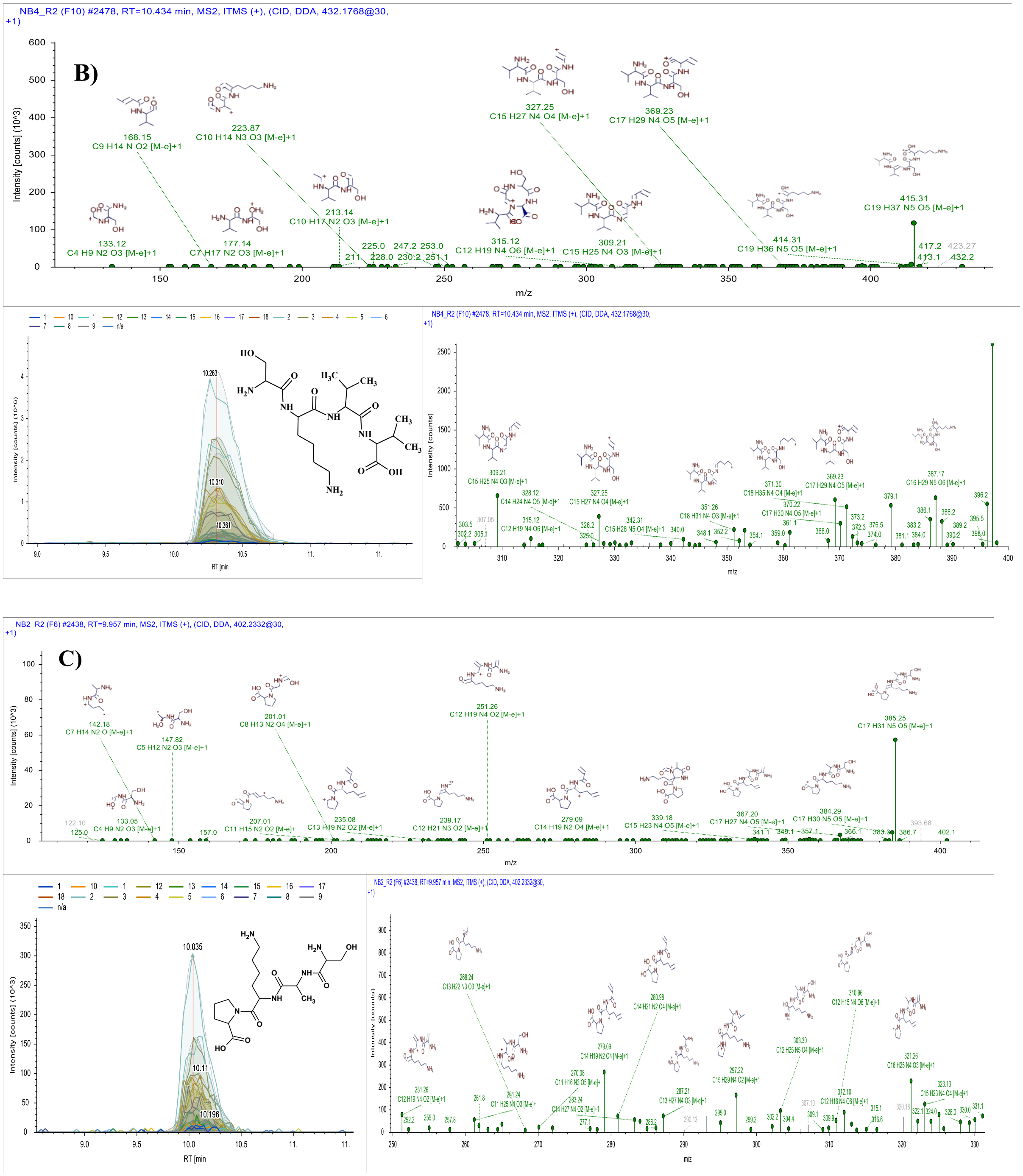

Figure S11. UPLC- OT-FTMS Chromatographic peak and FiSh fragmentation spectrum of A) Peptide Lys-Lys-Pro with chemical formula $C_{17}H_{33}N_5O_4$, FiSh fragmentation scan and peak B) Peptide Val-Val-Lys-Ser with chemical formula $C_{17}H_{31}N_5O_6$, C) Peptide Pro-Lys-Ala-Ser with chemical formula $C_{19}H_{37}N_5O_6$.

Supplement Tables

Table S1. A list of unlabeled organic acid standards and their mass error results with and without "lock mass" run on ion chromatography and negative mode detection.

No.	Unlabeled Organic Acid Standard	m/z $[M-H]^-$	No Lock Mass (ppm)	Lock Mass (ppm)	Retention Time (min)
1	Sodium isobutyrate	87.0452	0.5	-0.5	9.17
2	Sodium butyrate	87.0452	0.6	-0.5	9.41
3	Oxalic acid, disodium salt	88.9880	0.6	-0.4	19.85
4	Sodium L-lactate	89.0244	0.6	-0.4	7.56
5	α-Ketobutyric acid, sodium salt	101.0244	0.7	-0.3	10.38
6	Malonic acid, disodium salt	103.0037	0.5	-0.5	12.53
7	Sodium D-3-hydroxybutyrate	103.0401	0.6	-0.4	7.46
8	Fumaric acid, disodium salt	115.0037	1.0	0.4	12.84
9	Maleic acid, disodium salt	115.0037	1.0	0.4	12.84
10	α-Ketoisovaleric acid, sodium salt	115.0401	1.1	0.4	10.82
11	Methylmalonic acid, disodium salt	117.0193	1.0	0.3	12.37
12	Succinic acid, disodium salt	117.0193	1.0	0.3	12.37
13	α-Ketoisocaproic acid, sodium salt	129.0557	0.8	0.1	12.27
14	Malic acid, disodium salt	133.0142	1.1	0.2	12.30
15	α-Ketoglutaric acid, disodium salt	145.0142	0.8	0.1	13.07
16	Adipic acid, disodium salt	145.0506	1.2	0.3	12.20
17	DL 2-Hydroxyglutarate, disodium salt	147.0299	1.1	0.2	12.16

Table S2. A list of unlabeled amino acid and small metabolite standards and their mass error results with and without "lock mass" run on liquid chromatography and positive mode detection; amino acid and small metabolite standard mixture (A9906-1ml, Millipore Sigma).

No.	Unlabeled Amino Acid and Small Metabolite Standard	m/z $[M+H]^+$	No Lock Mass (ppm)	Lock Mass (ppm)	Retention Time (min)
1	L-Creatinine	114.0662	0.6	0.3	2.02
2	Taurine	126.0219	1.2	0.2	2.13
3	Hydroxy-L-proline	132.0655	1.1	0.2	2.13
4	L-Isoleucine	132.1019	1.2	0.2	3.09
5	L-Leucine	132.1019	1.2	0.2	3.09
6	L-Ornithine	133.0972	0.8	0.7	1.85
7	L-Aspartic acid	134.0448	1.4	0.0	2.33
8	L-Lysine	147.1128	1.0	0.0	1.89
9	L-Methionine	150.0583	1.5	0.0	2.75
10	L-Histidine	156.0768	1.4	0.1	1.82
11	δ-Hydroxylysine	163.1077	1.0	0.3	1.78
12	L-Phenylalanine	166.0863	1.5	-0.1	4.53
13	1-Methyl-L-histidine	170.0924	1.5	0.1	1.92
14	3-Methyl-L-histidine	170.0924	1.5	0.1	1.92
15	L-Arginine	175.1190	1.2	0.2	1.85
16	L-Citrulline	176.1030	1.4	0.0	2.09
17	L-Tyrosine	182.0812	1.5	0.1	2.71
18	L-Tryptophan	205.0972	1.5	-0.1	7.75
19	Cystathionine	223.0747	1.5	-0.1	1.96
20	L-Carnosine	227.1139	1.5	-0.2	1.82
21	L-Cystine	241.0311	1.5	-0.1	1.96
22	L-Anserine	241.1295	0.9	-0.2	1.92

Table S3. A list of unlabeled peptide standards and their mass error results with and without "lock mass" run on liquid chromatography and positive mode detection; UPLC peptide standard mixture (H2016, Sigma Aldrich).

No.	Unlabeled Peptide Standard	m/z $[M+H]^+$	No Lock Mass (ppm)	Lock Mass (ppm)	Retention Time (min)
1	Gly-Tyr	239.1026	1.6	-0.1	3.31
2	VAL-TYR-VAL	380.2180	1.8	-0.1	9.51
3	Leucine Enkephalin	556.2766	1.1	-0.1	12.55
4	Methionine Enkephalin Acetate	574.2330	0.8	0.3	11.63

References Cited

(1) Abdulla, H.A.; Sleighter, R.L.; Hatcher, P.G. Two dimensional correlation analysis of Fourier transform ion cyclotron resonance mass spectra of dissolved organic matter: A new graphical analysis of trends. *Analytical chemistry* **2013**, *85*(8), 3895-3902.

(2) Abdulla, H.A.; Burdige, D.J.; Komada, T. Accumulation of deaminated peptides in anoxic sediments of Santa Barbara Basin. *Geochimica et Cosmochimica Acta* **2018**, *223*, 245-258.

(3) Abdulla, H.A.; Burdige, D.J.; Komada, T. Abiotic formation of dissolved organic sulfur in anoxic sediments of Santa Barbara Basin. *Organic Geochemistry* **2020**, *139*, 103879.

(4) Douglas, A.R.; Murgulet, D.; Abdulla, H.A. Impacts of hydroclimatic variability on surface water and porewater dissolved organic matter in a semi-arid estuary. *Marine Chemistry* **2021** *235*, 104006.

(5) Kind, T.; Fiehn, O. Seven Golden Rules for heuristic filtering of molecular formulas obtained by accurate mass spectrometry. *BMC bioinformatics* **2007**, *8*(1), 1-20.

(6) Koch, B.P.; Dittmar, T.; Witt, M.; Kattner, G. Fundamentals of molecular formula assignment to ultrahigh resolution mass data of natural organic matter. *Analytical Chemistry* **2007**, 79(4), 1758-1763.
